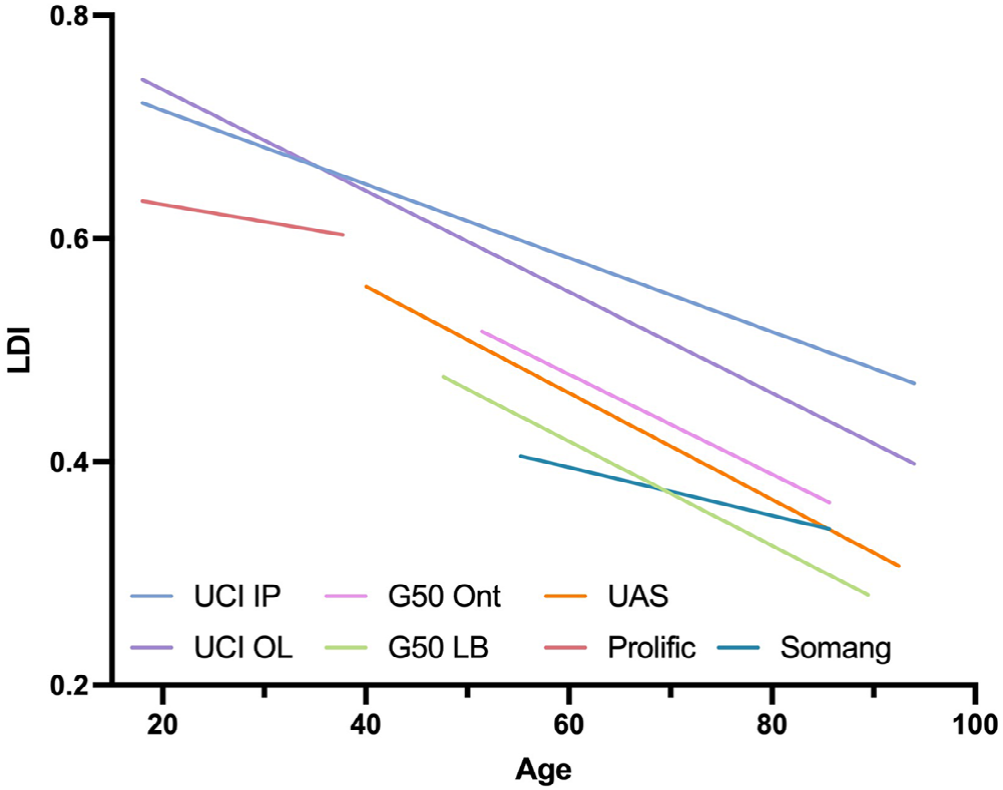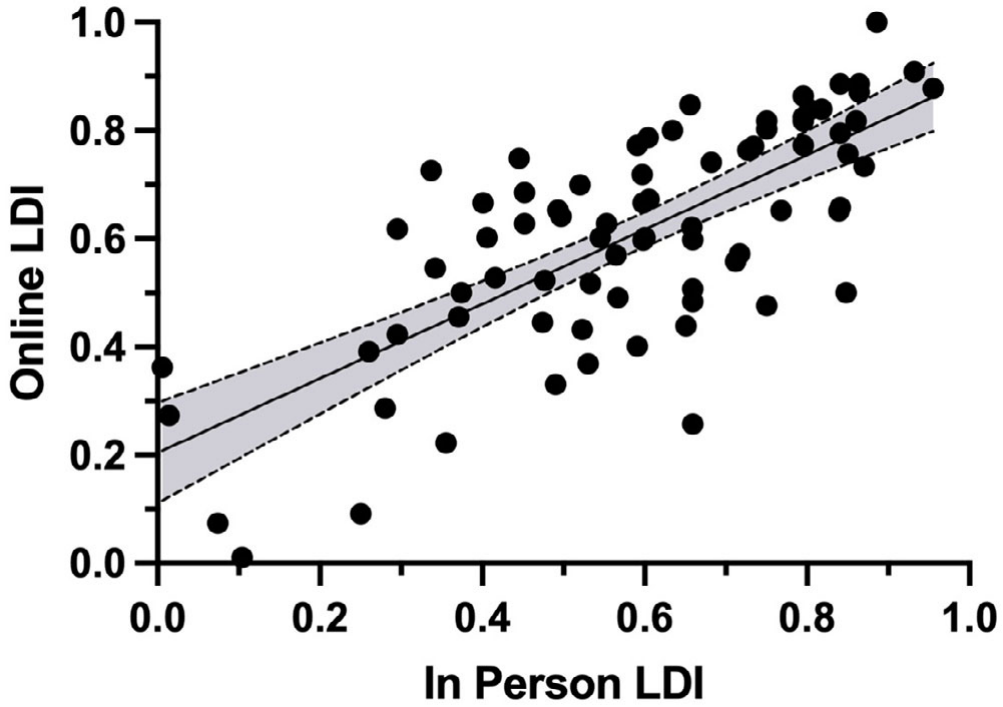# MST in the Wild: Optimizing the Mnemonic Similarity Task for Use in Diverse Environments

**DOI:** 10.1002/alz70858_101259

**Published:** 2025-12-25

**Authors:** Lilian Azer, Casey Vanderlip, Lizabeth L. Mayer, Luke Ehlert, Hye‐Won Shin, Craig EL Stark

**Affiliations:** ^1^ University of California, Irvine, Irvine, CA, USA; ^2^ Somang Society, Cypress, CA, USA; ^3^ RIIID Medical Group of Irvine, Irvine, CA, USA; ^4^ The UC Irvine Institute for Memory Impairments and Neurological Disorders, Irvine, CA, USA; ^5^ Department of Neurobiology and Behavior, University of California, Irvine, Irvine, CA, USA

## Abstract

**Background:**

Clear guidelines and tools for reliable measures of cognitive decline have yet to be established. Consequently, one‐third of Medicare Plan B beneficiaries received required cognitive screening during annual wellness visits (Jacobson et al., 2020). This gap may be due to the absence of consistent, easily administered, and scored screening tools.

**Method:**

Previously, we adapted the Mnemonic Similarity Task (MST; Stark et al., 2019), a research tool sensitive to hippocampal function and cognitive decline, into the optimized MST (oMST; Stark et al., 2023). The oMST is an automated, self‐administered, and scored measure designed for cognitive screening and population enrichment in clinical settings and at‐home use, offering a superior alternative to traditional neuropsychological tests. Here, we tested the oMST's reliability, validity, and accessibility across six experiments and 1,479 participants. Data was collected from UCI (in‐person, online, and ADRC), senior expos in California, Somang Korean Society, Understanding America Study, and Prolific.

**Result:**

Results showed strong test‐retest reliability, with lure discrimination highly correlated between in‐person and remote administration. Additionally, these results were consistent across diverse testing sites, demonstrating the oMST's robustness. Importantly, visual acuity, which may decline during normal aging, did not impact oMST performance.

**Conclusion:**

The oMST shows diagnostic potential in detecting subtle cognitive changes through cognitive modeling and may predict Alzheimer's disease biomarkers before noticeable decline (Vanderlip et al., 2024). Our findings further establish the oMST as a reliable and accessible tool for cognitive screening across diverse testing environments and administration methods, addressing critical gaps in early screening for cognitive decline.